# AlN Surface Passivation of GaN-Based High Electron Mobility Transistors by Plasma-Enhanced Atomic Layer Deposition

**DOI:** 10.1186/s11671-017-2082-0

**Published:** 2017-04-27

**Authors:** An-Jye Tzou, Kuo-Hsiung Chu, I-Feng Lin, Erik Østreng, Yung-Sheng Fang, Xiao-Peng Wu, Bo-Wei Wu, Chang-Hong Shen, Jia-Ming Shieh, Wen-Kuan Yeh, Chun-Yen Chang, Hao-Chung Kuo

**Affiliations:** 1 Department of Electrophysics, National Chiao Tung University, Hsinchu, 30010 Taiwan; 2 National Nano Device Laboratories, No. 26, Prosperity Road 1, Hsinchu, 30078 Taiwan; 3 Department of Photonics and Institute of Electro-Optical Engineering, National Chiao Tung University, Hsinchu, 30010 Taiwan; 4 NCTU-Picosun Joint Laboratories, National Chiao Tung University, Hsinchu, 30010 Taiwan; 5 International College of Semiconductor Technology, National Chiao Tung University, Hsinchu, 30010 Taiwan; 6 Research Center for Applied Sciences, Academia Sinica, 128 Academia Road, Section 2, Nankang, Taipei 11529 Taiwan

**Keywords:** GaN, High electron mobility transistor (HEMT), Atomic layer deposition (ALD), Current collapse, Surface passivation

## Abstract

We report a low current collapse GaN-based high electron mobility transistor (HEMT) with an excellent thermal stability at 150 °C. The AlN was grown by N_2_-based plasma enhanced atomic layer deposition (PEALD) and shown a refractive index of 1.94 at 633 nm of wavelength. Prior to deposit AlN on III-nitrides, the H_2_/NH_3_ plasma pre-treatment led to remove the native gallium oxide. The X-ray photoelectron spectroscopy (XPS) spectroscopy confirmed that the native oxide can be effectively decomposed by hydrogen plasma. Following the in situ ALD-AlN passivation, the surface traps can be eliminated and corresponding to a 22.1% of current collapse with quiescent drain bias (*V*_DSQ_) at 40 V. Furthermore, the high temperature measurement exhibited a shift-free threshold voltage (*V*_th_), corresponding to a 40.2% of current collapse at 150 °C. The thermal stable HEMT enabled a breakdown voltage (BV) to 687 V at high temperature, promising a good thermal reliability under high power operation.

## Background

Recent progress in high-power field-effect transistors (FET) was focused on GaN-based wide band-gap semiconductors. GaN-based high electron mobility transistors (HEMTs) have demonstrated a great potential due to their high breakdown electric field, low on-state resistance (*R*_on_), and high thermal stability [1, 2]. Therefore, GaN-based HEMTs provide significantly better performance compared with traditional Si-based power devices. However, GaN-based HEMTs meet the demand of reduction in dynamic on-resistance, which is so-called “current collapse” phenomenon during the high power switching. Current collapse phenomenon can be attributed to the high density of traps in GaN-based materials. The traps capture electrons and then act as a virtual gate on the surface, which deplete channel electrons and increase on-resistance simultaneously [3]. Previous studies suggest kinds of dielectric layer can be effective passivation layers, such as plasma-enhanced chemical vapor deposition (PECVD) grown SiN_x_ [4], atomic layer deposition (ALD) grown Al_2_O_3_ high-κ dielectric layer [5], and plasma-enhanced ALD (PEALD) grown AlN [6]. The effective passivation of PEALD grown AlN with in situ low-damage plasma pre-treatment enables to remove the surface native oxide with minimum surface damage. The surface native oxide is strongly related to the surface defects, leading to the current collapse and unreliable device performance [6]. Therefore, an optimum passivation layer with surface oxide removal process is a key technology to fabricate reliable GaN-based HEMTs. In this paper, we demonstrate a high reliable GaN-based HEMT regarding the PEALD-AlN passivation. Prior to AlN deposition, the hydrogen plasma carried out the surface native oxide removal process. The hydrogen plasma promised a low dynamic on-resistance, revealing a 22.1% of current collapse with quiescent drain bias (*V*_DSQ_) at 40 V. Moreover, the ALD-AlN with in situ plasma pre-treatment showed a 687 V of high breakdown voltage (BV) at 150 °C, promising a good thermal reliability under high power operation.

## Methods

### Epi-Wafers

**Fig. 1:**
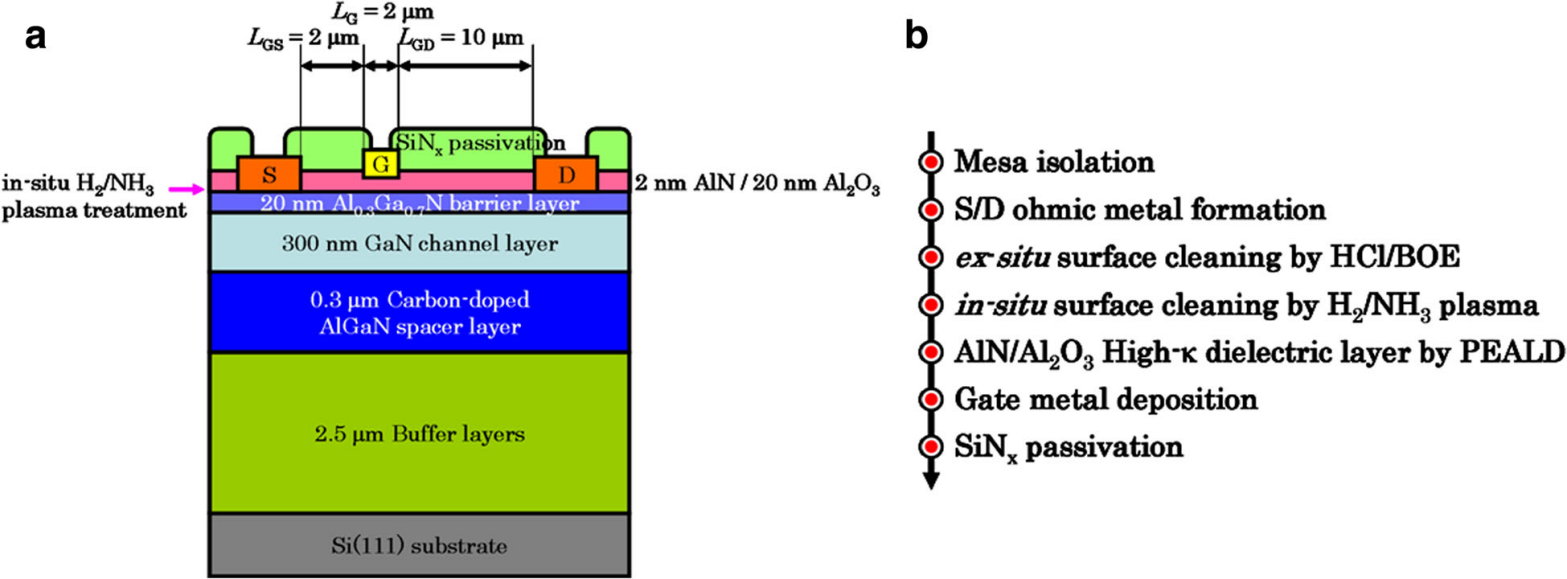
**a** Schematic cross section and dimensions of the HEMTs. **b** Device fabrication process flow

Al_0.3_Ga_0.7_N/GaN HEMT structure was grown on 6-inch Si(111) by low pressure metal-organic chemical vapor deposition (MOCVD) system. The HEMT structure exhibited two-dimensional electron gas (2DEG) sheet density of 1.07 × 10^13^ cm^−2^, 2DEG mobility of 1315 cm^2^/V•s, and sheet resistance of 447 Ω/sq. Figure 1a shows the structure diagram of epi-layers. However, GaN-on-Si with low-resistivity substrate tends to leak current from the channel to substrate, resulting in a reduction of BV [7]. We reported a low carbon-doped AlGaN back barrier, which showed a comparable BV to that of regular devices, but the trap density can be minimized or eliminated due to its higher growth temperature [8].

### Device Fabrications

The epi-wafers were separated into pieces by 2 × 2 cm^2^. First, the BCl_3_/Cl_2_-based inductively coupled plasma etching was employed to define the mesa isolation. The specific contact resistance (*ρ*_c_) of 2.34 × 10^−6^ Ω-cm^2^ was obtained by Ti/Al/Ni/Au (25/125/40/150 nm) ohmic metal and thermal metallization by a rapid thermal annealing (RTA) at 850°C for 30 sec. Before passivation, ex situ surface cleaning by HCl:DI = 1:1 and BOE:DI = 1:100 was carried out the residual carbon and native oxide removal. Afterward, the surface was secondly treated by remote plasma by *Picosun* ^*TM*^ R200 PEALD. Next, the high-κ insulator was in situly deposited. After the gate insulator was deposited, the Ni/Au (50/200 nm) Schottky gate metal was evaporated. The HEMT device was realized with a 2-μm gate length (*L*_G_), 2-μm source-to-gate distance (*L*_SG_), 10-μm gate-to-drain distance (*L*_GD_), and 100-μm gate width (*W*_G_). Finally, a 150-nm-thick SiN_x_ was deposited by plasma-enhanced chemical vapor deposition (PECVD) at 300 °C. The process flow is presented in Fig. 1b.

### Surface Pre-Treatment and AlN Passivation

AlN-passivated HEMT received an identical in situ cleaning process by 1500 W H_2_/NH_3_ remote plasma pre-treatment. The pre-treatment process was consisted by 36 cycles of digital H_2_/NH_3_ plasma for 5 s/5 s of pulse/purge time. Following the surface pre-treatment, the stacking 2-nm-thick AlN and 20-nm-thick Al_2_O_3_ were grown on the HEMT surface to serve as gate insulator. The ALD-AlN was employed to isolate oxygen into GaN-based materials for preventing the formation of Ga-O compound [6]. In this study, we fabricated two growth conditions of AlN. First AlN was grown at 350 °C in Ar ambient by applying 0.1 s 99.999% pure trimethylaluminum (TMA), and 5.5 s long, 2800 W plasma-activated N_2_ pulses. Each pulse was separated by purge steps with 99.999% pure Ar only flowing for 8 s. Second ALD-AlN was grown at 300 °C, 0.1 s TMA and 11.5 s long, 2800 W plasma-activated NH_3_ pulses. The purge time was kept at 8 s via Ar purging. Afterwards, the 20-nm thick thermal ALD-grown Al_2_O_3_ was in situly grown on AlN. The Al_2_O_3_ was grown at 300 °C in Ar ambient by applying 0.1 s TMA, and 0.1 sec for H_2_O through 7 s Ar purging. The growth parameters are listed in Table 1.

**Table 1:** Surface treatment and high-κ dielectric growth condition by PEALD

Condition	TMA		2800 W N_2_ Plasma (Ar: 160 sccm)		2800 W NH_3_ Plasma (Ar: 110 sccm)		1500 W H_2_ Plasma (Ar: 30 sccm)		1500 W NH_3_ Plasma (Ar: 30 sccm)		Cycles
	Flow	Pulse/purge	Flow	Pulse/purge	Flow	Pulse/purge	Flow	Pulse/purge	Flow	Pulse/purge	
Pre-treatment (300 °C)							15	5/5	50	5/5	36
N_2_-based AlN (350^o^C)	150	0.1/5	40	5.5/8							45
NH_3_-based AlN (300 °C)	150	0.1/5			80	11.5/8					19
Unit:	(sccm)	(s)	(sccm)	(s)	(sccm)	(s)	(sccm)	(s)	(sccm)	(s)	cyls

The X-ray photoelectron spectroscopy (XPS) using a PHI QuanteraII system was employed to determine the stoichiometry of the dielectric films. The narrow scan mode with Ar sputtering investigated the depth profile of the elemental composition during the interface. The minimum spot size is 7.5-um-diameter. The J.A. Woollam M-2000 spectroscopic ellipsometer was used to determine the refractive index (n) spectrum of AlN. The deuterium light source with the minimum beam size is 0.3 mm.

## Results and Discussion

**Fig. 2:**
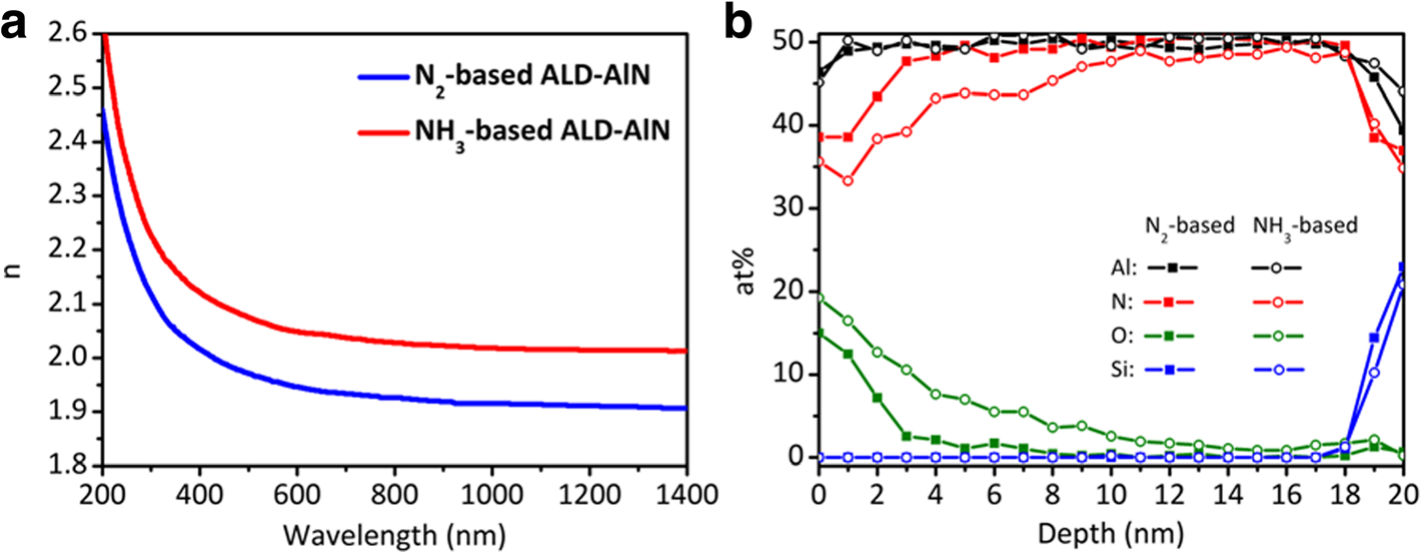
**a** Refractive index as a function of the wavelength. **b** XPS depth profiles of the AlN films grown by N_2_ (*closed square*) and NH_3_ (*open circle*) ambient plasma. The ALD-AlN was grown on Si(100) substrate

The refractive index spectrum of ALD-AlN is shown in Fig. 2a. The ALD-AlN was grown on Si(100) substrate to investigate the optical characteristics. The refractive index shows 1.94 and 2.04 at 633 nm of wavelength for N_2_-based and NH_3_-based AlN, respectively. In previous studies, the refractive index of AlN was reported by 2.09 at *λ* = 632.8 nm, which was grown by MOCVD [9]. In addition, the radio frequency magnetron sputtering grown AlN suggested the refractive index is between 1.95 and 2.05 at 633 nm [10]. A lower refractive index can be observed from the PEALD grown AlN films, which can be attributed to a lower crystallinity of PEALD-grown AlN. S. Huang et al. reported that regional polycrystalline domains appeared at the top of the ALD-grown AlN layer, which were responsible for the lower refractive index. However, the single crystal AlN can be grown in the first mono-layers, leading to a good surface passivation for GaN-based HEMTs [6]. Indeed, the MOCVD-grown AlN shows a higher refractive index than ALD grown AlN but the fact that we cannot growth AlN on GaN-on-Si based HEMT structure without cracks [11]. Also, sputtering AlN tends to damage the surface due to the ion bombardment by plasma [12]. As can be seen, the ALD-grown AlN with comparable refractive index is the best choice for GaN-based HEMTs. Moreover, the NH_3_-based ALD-grown AlN shows a higher refractive index than N_2_-based AlN but the growth rate of NH_3_-based AlN is almost two times faster than N_2_ ones. The XPS depth profile investigation suggests higher oxygen content in NH_3_-based AlN thin film, as shown in Fig. 2b. The higher oxygen content is most likely caused by sample exposed to atmosphere as well as leaked oxygen into the film. The feature suggests that the ALD-AlN with higher growth rate is not compact enough than N_2_-based ALD-AlN. Therefore, the N_2_-based ALD-AlN will be the first choice of surface passivation for GaN-based HEMTs.

**Fig. 3:**
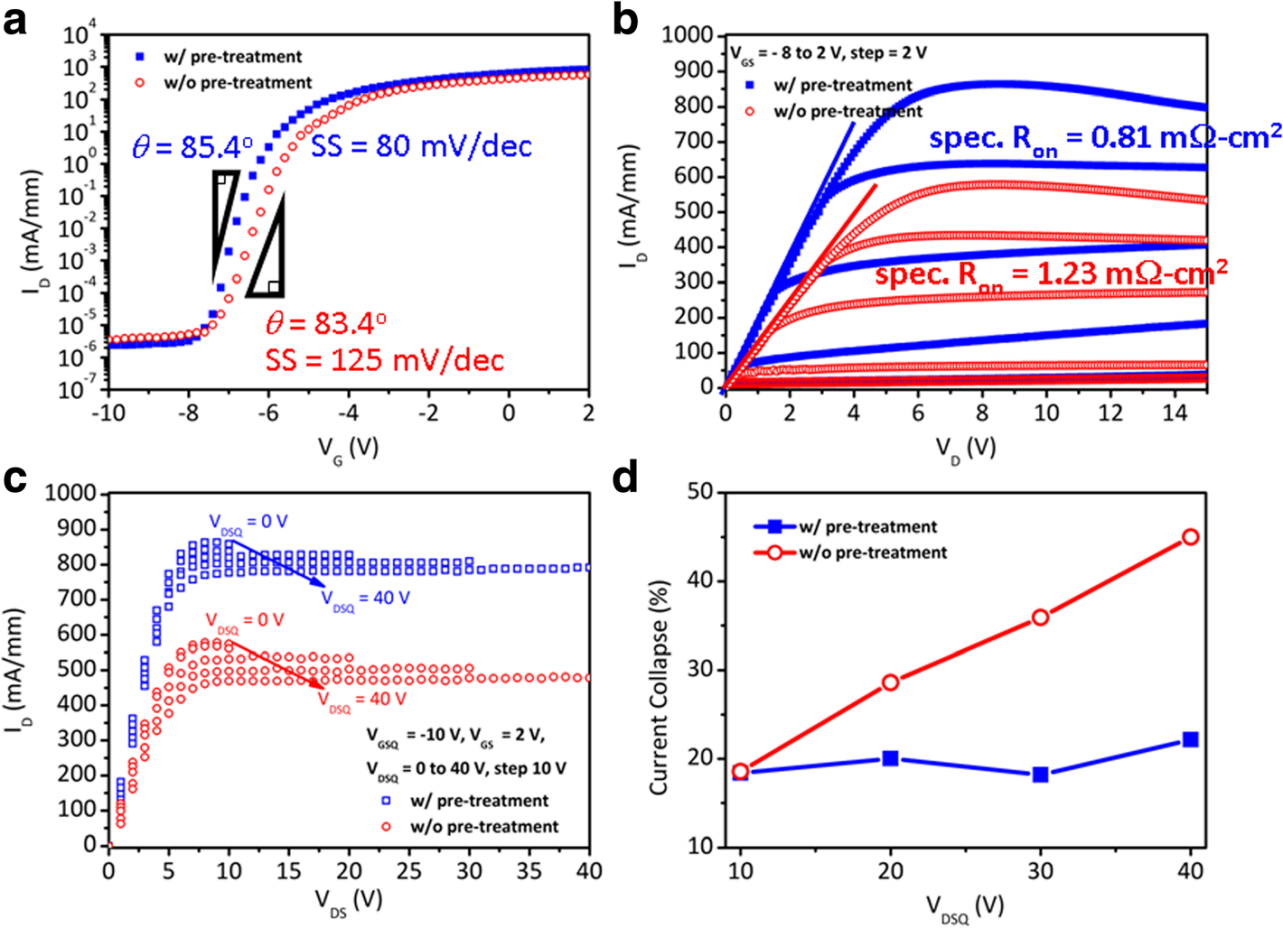
**a** *I*_DS_-*V*_GS_ characteristics of the ALD-AlN passivated HEMT with (*blue squares*) and without (*red circles*) plasma pre-treatment (*V*_DS_ = 10 V). **b** *I*_DS_-*V*_DS_ characteristics of ALD-AlN passivated HEMT (*V*_GS_ from -8 to 2 V with 2 V step). **c** Pulsed *I*_DS_-*V*_DS_ characteristic of HEMT with (*blue squares*) and without (*red circles*) plasma pre-treatment, *V*_GSQ_ at -10 V, *V*_DSQ_ sweep from 0 V to 40 V. **d** Current collapse (%) versus quiescent drain bias (*V*_DSQ_)

The DC characteristics of *I*_DS_-*V*_GS_ (*V*_DS_ = 10 V) for ALD-AlN passivated GaN-based HEMTs are shown in Fig. 3a. The ALD-AlN passivated HEMTs reveal the gate modulation and pinch-off characteristics. The maximum drain currents (*I*_DS,max_) are 832 and 573 mA/mm for the AlN-passivated HEMT with plasma pre-treatment and without pre-treatment, respectively. Figure 3b shows the *I*_DS_-*V*_DS_ characteristics of the HEMTs. The drain current of the AlN-passivated HEMT without plasma pre-treatment shows 577 mA/mm at 2 V of gate bias (*V*_GS_). The characteristic of AlN-passivated HEMT with plasma pre-treatment is comparatively enhanced, which reveals a higher drain current of 863 mA/mm. The specific on-resistance (*spec.* R_on_) of the AlN-passivated HEMTs are 0.81 and 1.23 mΩ-cm^2^ for plasma pre-treatment HEMT and without pre-treatment one, respectively.

The values of *I*_DS,max_ for the HEMT with plasma pre-treatment were relatively high regarding the mobility enhancement due to surface passivation eliminated carrier scattering [13, 14]. The better performance implies that the H_2_/NH_3_ plasma-pretreatment leads to a high quality interface between ALD-AlN and III-nitrides. To further investigate the gate control characteristics for both devices, the subthreshold swing (SS) is a parameter which clearly indicates the interface quality. The SS is defined to be the inverse slope of the log (*I*_DS_) versus *V*_GS_ characteristic in the subthreshold region. The values of SS are 80 and 125 mV/dec for the HEMT with and without plasma pre-treatment, respectively. The lower SS value confirms a lower interfacial state density after plasma pre-treatment. The interfacial states can be attributed to the native Ga-O bonds, which result in a high density of surface traps. Hence, it is essential to remove the native oxide on HEMTs surface. The hydrogen plasma enables to remove native oxide on the GaN surface, following the ammonia plasma will passivate the surface by plasma nitridation simultaneously. In addition, surface nitridation also isolates the Ga and O atoms, preventing the formation of Ga-O bonds.

The pulsed *I*_DS_-*V*_DS_ characteristics were extracted from the off-state with a quiescent gate bias (*V*_GSQ_) of -10 V to the on-state at 2 V in 500 ns and a separation of 1 ms. Then, the quiescent drain bias (*V*_DSQ_) was swept from 0 V to 40 V (10 V step). As a result for plasma pre-treated HEMT, Fig. 3c shows a smaller dispersion of *I*_DS_-*V*_DS_ after H_2_/NH_3_ plasma pre-treatment, thereby surface trap was successfully decreased. The current collapse was investigated and summarized in Fig. 3d, which shows a smaller discrepancy after ALD H_2_/NH_3_ plasma pre-treatment. The current collapse was defined as:

$$ \frac{R_{on, dynamic}-{R}_{on, static}}{R_{on, static}}\times 100\% $$

The current collapse was 22.1% for the HEMT with plasma pre-treatment but the absent of pre-treatment HEMT shows 44.9% at *V*_DSQ_ = 40 V. The current collapse phenomenon can be effectively reduced by plasma pre-treatment for the GaN-based HEMTs.

The chemical properties of HEMT surface was clearly investigated by XPS spectroscopy. The XPS measurement system consists of a spherical capacitor analyzer and a monochromated Al K_α_ x-ray source (hν = 1486.6 eV). The binding energies of the spectra were carefully calibrated through separated measurements of Cu 2p_3/2_, Ag 3d_5/2_, and Au 4f_7/2_ peak positions. The XPS spectroscopy enables to verify the native oxide was removed by plasma pre-treatment process, leading to a high quality interface and promised low current collapse. The Ga_2_O_3_ can be decomposed by hydrogen plasma as [15, 16]:

$$ {\mathrm{Ga}}_2{\mathrm{O}}_3 + 4\mathrm{H}*\ \to\ \left(\ {\mathrm{Ga}}_2\mathrm{O}\uparrow + 2{\mathrm{H}}_2\mathrm{O}\uparrow \right) + \left(\ 2\mathrm{GaOH}\uparrow + {\mathrm{H}}_2\mathrm{O}\uparrow \right) $$

**Fig. 4:**
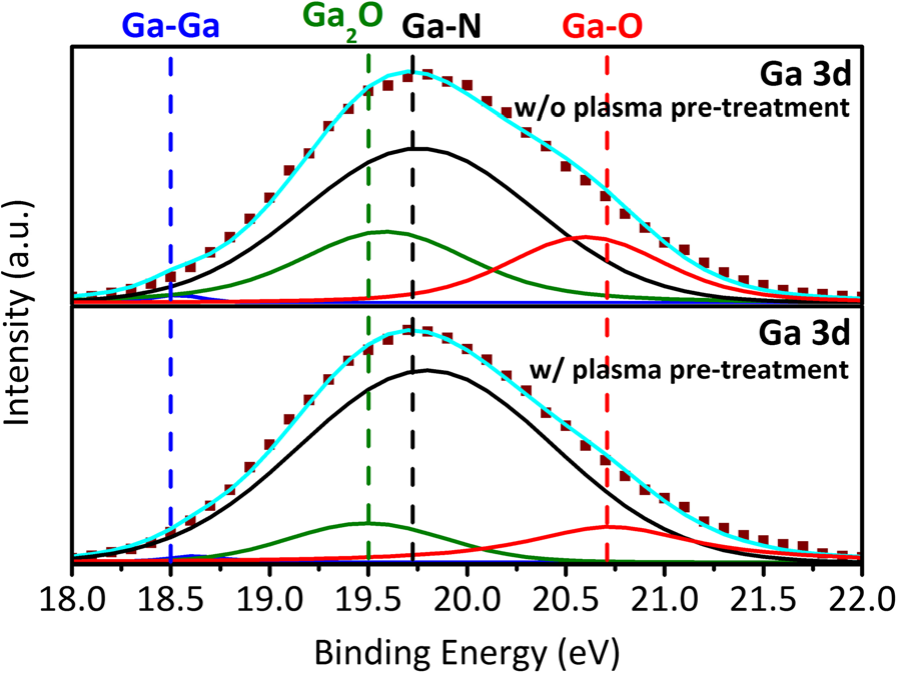
XPS spectroscopy of Ga 3d peak at the interface between AlN and AlGaN for HEMTs without (Upper) and with (under) plasma pre-treatment. The spectrum was fitted by Gaussian function and separated by four major components (*solid lines*) corresponding to Ga-Ga (*blue*), Ga_2_O (*green*), Ga-N (*black*), and Ga_2_O_3_ (*red*)

Figure 4 shows the Ga 3d core-level spectra of the two samples. The peaks can be realized by four major peaks to Ga-Ga at 18.5 ± 0.1 eV, Ga^+^-O (Ga_2_O) at 19.5 ± 0.1 eV, Ga-N at 19.7 ± 0.1 eV, and Ga^3+^-O (Ga_2_O_3_) at 20.7 ± 0.1 eV [17]. The two oxidation states of Ga_2_O and Ga_2_O_3_ contributed to the Ga-O bonds. The Ga-O/Ga-N bond ratio provides a clearly evidence for the native oxides were removed. The Ga-O/Ga-N bond ratio was reduced from 33.1 to 17.8% as we adopted plasma pre-treatment, implying that the native Ga_2_O_3_ was effectively eliminated by H_2_/NH_3_ plasma. Consequently, the surface traps can be significantly reduced, which is consisted to the pulsed *I*_DS_-*V*_DS_ investigation results.

**Fig. 5:**
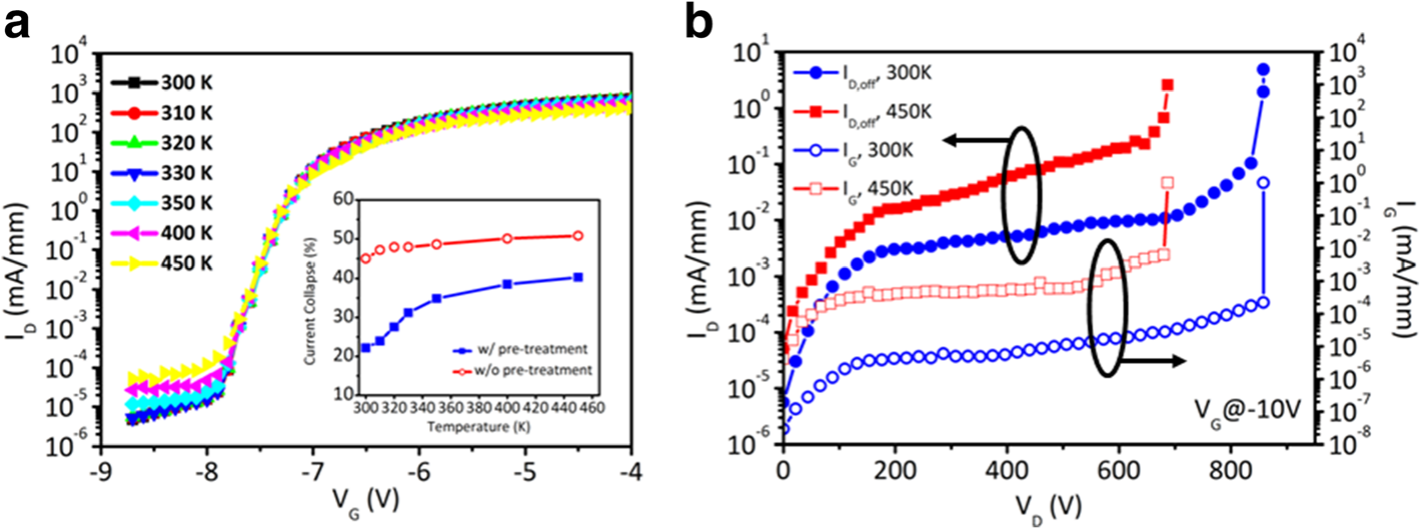
**a** Temperature dependent *I*_DS_-*V*_GS_ of plasma pre-treated HEMT. The *inset* shows the current collapse with increased temperature. **b** The temperature dependent BV measurement of plasma pre-treated HEMT. The *void lines* show the temperature dependent *I*_GS_ of plasma pre-treated HEMT

However, the demands of GaN-based HEMTs require a thermal stability as high-voltage power switching devices typically operate at elevated junction temperature (*T*_j_). It's a difficult task for GaN-based HEMTs operate at high *T*_j_ in particular a significant negative shift of threshold voltage (*V*_th_) via high density traps present and were thermally activated at the interface between dielectric and GaN-based materials [18]. The temperature-dependent *I*_DS_-*V*_GS_ of the plasma pre-treated HEMT was plotted in Fig. 5a. The off-state *I*_DS_ increased 2 orders of magnitude when the measured temperature increased from room temperature to 150 °C. The raised off-state *I*_DS_ can be attributed to increased buffer leakage [19]. As can be seen, a nearly shift-free of *V*_th_ was obtained from RT to 150 °C, only 0.02 V negative shifting of *V*_th_ was observed. The shift-free of *V*_th_ indicates the surface negative charges were eliminated. Previous studies suggest a thermally induced negative *V*_th_ shift is 0.5 V [19]; hence, the ALD AlN-passivated HEMTs with plasma pre-treatment proves that the trap density can be effectively improved. The inset of Fig. 5a summarizes the measurement results of current collapse under elevated temperatures, where the current collapse results were measured at *V*_DSQ_ = 40 V. A clear suppression of current collapse was observed at higher temperatures for the HEMT with plasma pre-treatment, only 40.2% of current collapse at 150 °C. In contrast, the current collapse was increased to 50.8% at 450K for the HEMT without plasma pre-treatment. The result can be attributed to suppression the influence of trap states by native oxide removal. The traps will capture electrons from the gate or 2DEG channel at lower temperatures but release trapped electrons at higher temperatures, leading to a thermal-induced *R*_on_ variation. In general, the surface traps will be fixed at positive charges regardless of temperature and bias by effective passivation. However, it is difficult to well-passivate high density of traps. The best way to improve thermal activated current collapse is still trying to overcome the high density of traps. Therefore, H_2_/NH_3_ plasma pre-treatment promised a low density of surface traps, corresponding to ALD-AlN passivation leads to lower negative surface charges.

Figure 5b shows the temperature dependent BV and gate leakage (*I*_GS_) for plasma pre-treated HEMT. The soft BV was defined as off-state *I*_DS_ at 1 μA. The soft BV was reduced from 660 to 153 V as we increased the temperature from RT to 150 °C. The device BV can be reached to 858 V at RT but decreased to 687 V at 150 °C. We should notice that the gate leakages suggest the BVs were limited by impact ionization, as shown in the figure with void lines. Despite the soft BV was reduced to 153 V, a thermal stable HEMT enabled a BV > 600 V at high temperature, promising a good thermal reliability under high power operation. The ALD approaches improve device performance and provide an effective and easy methods for preventing undesirable phenomena, producing reliable devices for high power applications.

## Conclusions

In summary, we fabricated a low current collapse GaN-based HEMT with an excellent thermal stability. The ALD-AlN shows a refractive index of 1.94 at 633 nm of wavelength. Prior to deposit AlN on III-nitrides, the H_2_/NH_3_ plasma pre-treatment resulted in a low trap density of the surface. The hydrogen plasma enables to effectively decompose native gallium oxide. The XPS spectroscopy reveals that the native oxide was removed. Following the in situ ALD-AlN leading to a well-passivated surface results in a low current collapse of 22.1% with quiescent drain bias (*V*_DSQ_) at 40 V. Furthermore, the high temperature measurement exhibited a shift-free of *V*_th_, corresponding to a 40.2% of current collapse at 150 °C. The thermal stable HEMT enabled a BV > 600 V at high temperature, promising a good thermal reliability under high-power operation.
